# SVCT2-GLUT1-mediated ascorbic acid transport pathway in rat dental pulp and its effects during wound healing

**DOI:** 10.1038/s41598-023-28197-9

**Published:** 2023-01-23

**Authors:** Naoto Ohkura, Kunihiko Yoshiba, Nagako Yoshiba, Naoki Edanami, Hayato Ohshima, Shoji Takenaka, Yuichiro Noiri

**Affiliations:** 1 Division of Cariology, Operative Dentistry and Endodontics, Department of Oral Health Science, Niigata University Graduate School of Medical and Dental Sciences, Niigata, Japan; 2 Division of Oral Science for Health Promotion, Department of Oral Health and Welfare, Niigata University Graduate School of Medical and Dental Sciences, Niigata, Japan; 3 Division of Anatomy and Cell Biology of the Hard Tissue, Department of Tissue Regeneration and Reconstruction, Niigata University Graduate School of Medical and Dental Sciences, Niigata, Japan

**Keywords:** Cell biology, Molecular biology

## Abstract

Ascorbic acid (AA; vitamin C) plays a crucial role in the biosynthesis and secretion of collagen to produce the organic matrix of hard tissues. Nevertheless, the detailed mechanism by which AA induces reparative dentinogenesis is still unknown. This study aimed to investigate the pathway and function of AA during wound healing in a rat pulpotomy model. Sodium-dependent vitamin C transporter (SVCT) 2 and glucose transporter (GLUT) 1 were detected in odontoblasts, endothelial cells, and nerve fibers in normal pulp tissues. SVCT2 and GLUT1 were also expressed in odontoblast-like cells in pulpotomized tissues of Wistar rats, and immunopositive cells of SVCT2 were significantly increased at 5 days after pulpotomy (*p* < 0.05). By contrast, osteogenic disorder Shionogi (ODS) rats, which cannot generate AA, also expressed SVCT2 and GLUT1 in normal and wound healing conditions. However, in ODS rats, when compared with the AA-addition group, the formation of dentin bridges in the AA-loss group was not evident, a layer of osteopontin was significantly increased beneath the wound surface (*p* < 0.05), and alpha smooth muscle actin at the odontoblast-like cells observed along this layer was significantly increased (*p* < 0.05), but not Nestin. Moreover, the amounts of type 1 collagen generated in the reparative dentin and beneath the wound healing site were significantly diminished (*p* < 0.05). Macrophages expressing CD68 and CD206 increased beneath the wound site. Hence, AA may be involved in odontoblast-like cell differentiation and anti-inflammatory response during dental pulp wound healing. Our results provide new insights into the function of AA through SVCT2 and GLUT1 in reparative dentinogenesis and may help in developing new therapeutic targets for dental pulpal disease.

## Introduction

Exposed dental pulp by progressed caries or traumatic injury often leads to infection. Consequently, exposed sites are covered by pulp capping materials by inducing the layer of reparative dentin^1^. Mineral trioxide aggregate (MTA), a calcium silicate-based cement, prevents bacterial infection and accelerates reparative dentin formation^2^. Therefore, many dentists often use MTA to protect the pulp tissue from bacteria. However, regarding pulp capping for humans, there is a time lag between MTA coverage to the exposed pulp sites and the formation of tertiary dentin to protect the wound site. This may raise the probability of bacterial infection. Thus, the construction of tertiary dentin is necessary as soon as possible after treatment.

Ascorbic acid (AA; vitamin C) plays an important role in the biosynthesis and secretion of collagen, the main component of the organic matrix of hard tissue. AA is biosynthesized from glucose in the liver in many mammals. However, humans and guinea pigs directly require dietary AA because of the hereditary lack of l-gulono-γ-lactone oxidase (GLO), which catalyzes the last response of the AA biosynthetic pathway. The lineage of osteogenically disordered Shionogi (ODS) rats was created from a spontaneous mutant of the Wistar rat^3^. ODS rats mutated by the GLO gene of Wistar rats cannot synthesize AA^4^. Therefore, ODS rats may help in investigate the in vivo efficacy of AA deficiency on the formation and physiological function of hard tissue.

A study reported that ODS rats induced bone loss and reduction during bone formation (change of bone conditions with AA deficiency in ODS rats). Similarly, several researchers have mentioned the role of AA during dentinogenesis in ODS rats. Another study reported that the predentin layer in the crown of ODS rat molars showed abnormal thickness, and almost all the roots are missing compared with normal rats^5^. Moreover, the predentin layers of ODS rats do not have collagen fibrils^5^. To our knowledge, no study has examined the histological or pathological mechanism of AA during the healing process in the dental pulp of ODS rats. In particular, data on the function of AA in reparative dentin formation after pulp capping are poor.

How is AA carried to various parts of our body? AA exists in two compounds, the reduced form (AA) and the oxidized form (dehydro-ascorbic acid; DHA)^6^. At physiological pH, AA is negatively charged, and DHA is a noncharged molecule although it is hydrophilic. Thus, specific transport pathways are involved in the transfer of both compounds through cell membranes, as lipophilic compounds can transport the transmembrane. AA is uptaken intracellularly by sodium-dependent vitamin C transporter (SVCT)1 and SVCT2. SVCT1 and SVCT2 are specific transporters for AA that are carried by the Na^+^ concentration gradient^7^. Moreover, SVCT2 has a higher affinity for AA than SCVT1^8^. By contrast, DHA has a similar molecule structure to glucose and is mainly released from the cells via GLUT1. These pathways of AA via SVCTs are present in the kidney, liver, and small intestine^9^. The GLUT1 of the DHA pathway is expressed in almost all tissues^10^. To our knowledge, no studies have examined AA and DHA pathway in dental pulp tissue.

As explained above, AA in exposed dental pulp tissue may be involved in the formation of reparative dentin during wound healing. We hypothesized that (1) the transporting pathways of AA via SVCT2 and GLUT1 exist in the dental pulp tissue and (2) AA mediates collagen formation and differentiation of odontoblast-like cells during wound healing.

Thus, our aim was to elucidate the expression pathway via AA transporters during wound healing in the pulp after MTA capping and assess the functional meaning of AA with immunohistochemistry using ODS rats. To the best of our knowledge, this is the first report to suggest that the SVCT2–GLUT1 AA pathway exists in rat dental pulp tissue, and our data showed that AA supplementation plays an important role in regulating dentinogenesis during wound healing. These new understandings may serve the development of dramatic therapy of exposed pulp tissue.

## Results

### Localization of AA pathways in normal rat molar pulp

**Figure 1:**
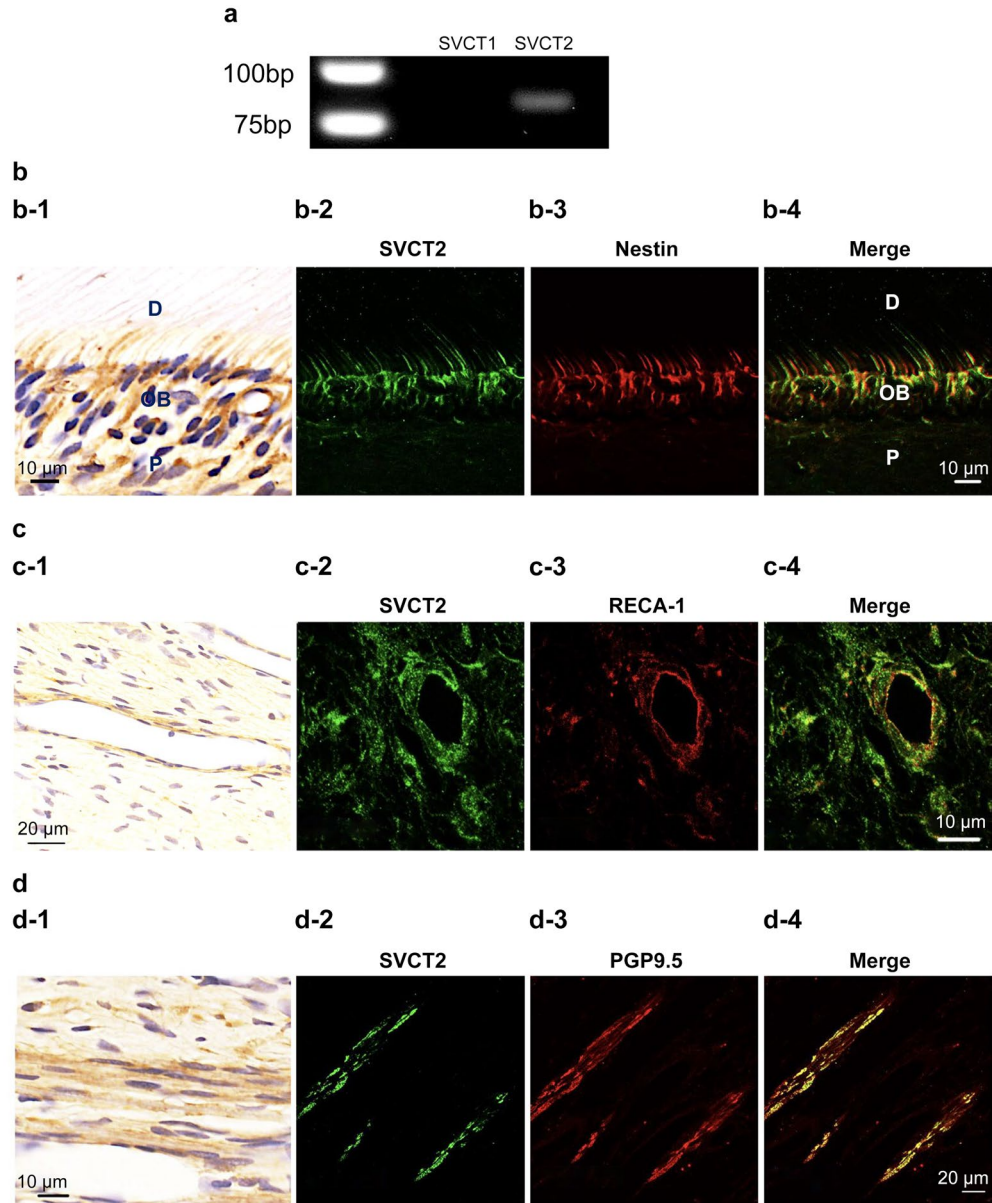
Localization of sodium-dependent vitamin C transporter 2 (SVCT2) immunoreactivity in normal pulp tissue. (**a**) Reverse transcription-polymerase chain reaction analysis. SVCT2 mRNA was expressed in the dental pulp tissue, but not SVCT1. (b-1, c-1, and d-1) Immunohistochemical staining of SVCT2 and double immunofluorescence staining of (b-2–b-4) SVCT2 and Nestin (odontoblast marker), (c-2–c-4) SVCT2 and RECA-1 (endothelial cells marker), and (d-2–d-4) SVCT2 and PGP9.5 (nerve fiber marker). (b-2, c-2, and d-2) SVCT2 staining (green). (b-3) Nestin staining (red). (c-3) RECA-1 staining (red). (d-3) PGP9.5 staining (red). (b-4, c-4, and d-4) Merged images. Immunoreactivities of SVCT2 were detected in odontoblast cytoplasm and processes as well as blood vessels and nerve fibers. D, dentin, OB, odontoblast, P, dental pulp.

**Figure 2:**
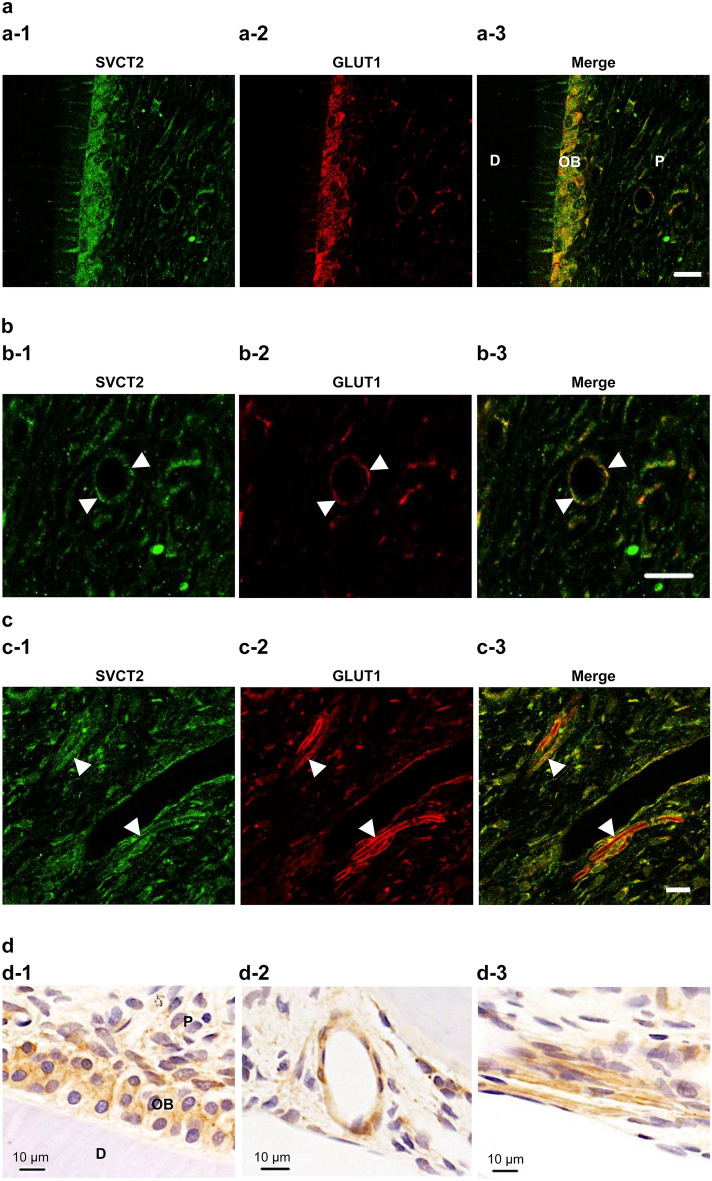
SVCT2 was colocalized in glucose transporter 1 (GLUT1) in odontoblasts, blood vessels, and nerve fibers in normal pulp tissue. Double immunofluorescence staining of (a-1, b-1, and c-1) SVCT2 (green) and (a-2, b-2, and c-2) GLUT1 (ted). (a-3, b-3, and c-3) Merged images. (b-1–b-3) The arrowheads indicate the blood vessel. (c-1–c-3) The arrowheads indicate the nerve fibers. (**d**) Immunochemical stating of GLUT1. D, dentin, OB, odontoblast, P, dental pulp. Scale = 20 μm.

As a first step toward understanding whether SVCT1 and SVCT2 are expressed in normal rat molar pulp, we performed the reverse transcription-polymerase chain reaction (RT-PCR). Consequently, SVST2 was only expressed in pulp tissues (Fig. 1a, the full-length gel is shown in Supplementary Fig. 1). Thus, we performed the immunohistochemical staining to confirm where SVCT2 is expressed in normal rat molar pulp. As shown in Fig. 1, double immunofluorescence staining demonstrated that the immunoreactivities of Nestin (an odontoblast marker, Fig. 1b), rat endothelial cell antigen-1 (RECA-1, endothelial cell marker, Fig. 1c), and PGP9.5 (peripheral nerve fibers, Fig. 1d) overlapped with that of SVCT2. Previously, we reported that GLUT1 was also expressed in odontoblasts, endothelial cells, and peripheral nerve fibers^11^. Thus, we hypothesized that SVCT2-positive cells were consistent with GLUT1-positive cells. Then, we confirmed these results by double immunofluorescence, which showed that SVCT2 immunoreactivity overlapped with that of GLUT1 in odontoblasts, endothelial cells, and peripheral nerve fibers (Fig. 2).

### Expression and mRNA alterations of SVCT2 in pulpotomized rat pulp tissue

**Figure 3:**
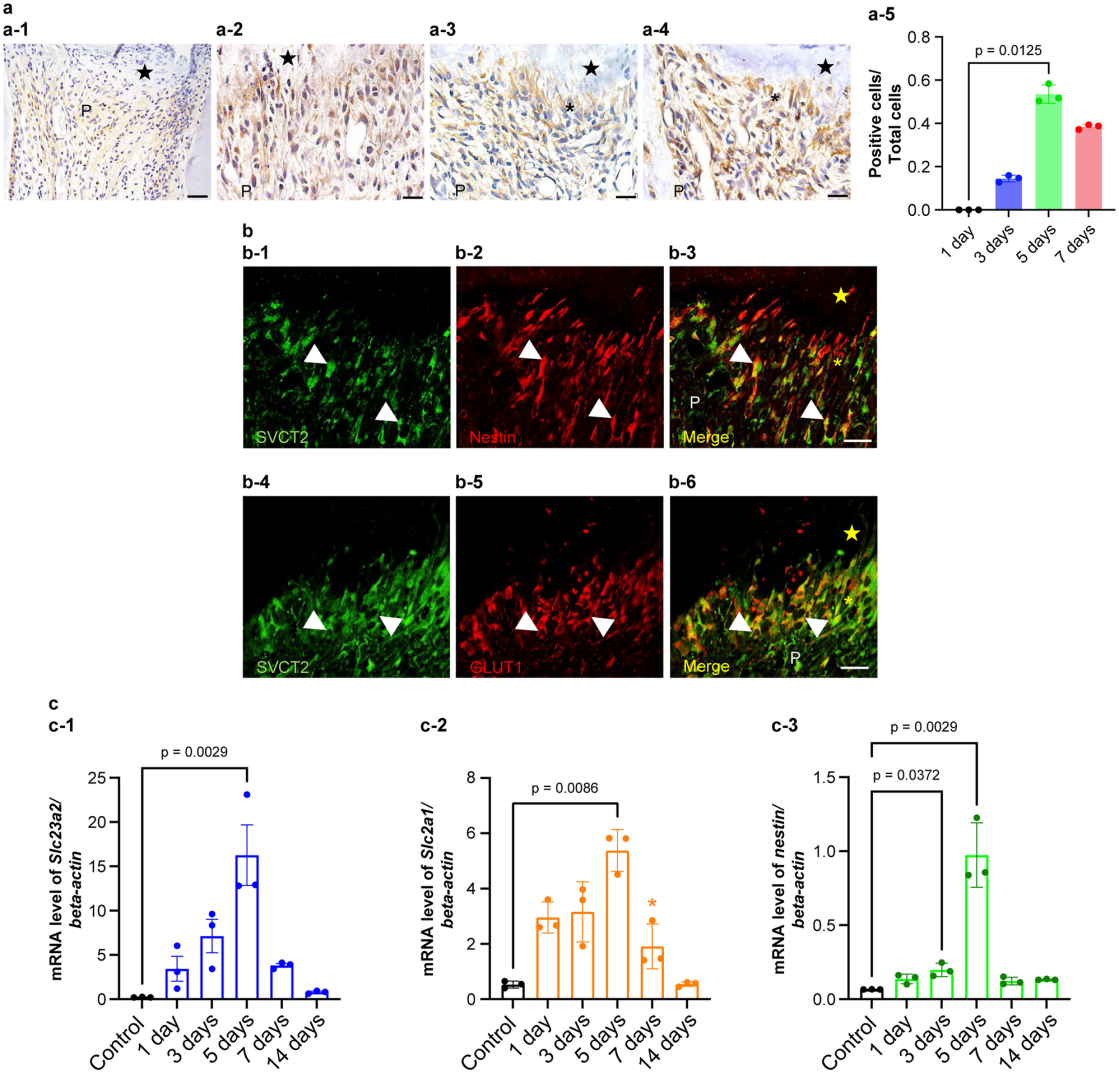
Alteration of SVCT2 expression over time in the pulp tissue after pulpotomy followed by MTA capping. (**a**) Immunohistochemistry of SVCT2 in the coronal pulp tissue of the injured teeth at (a-1) 1, (a-2) 3, (a-3 and b-1–b-6) 5, and (a-4) 7 days after pulpotomy (n = 3 at each time point). (a-2) At 3 days after pulpotomy, immunoreactivities of SVCT2 were seen throughout the pulp tissue. Reparative dentin initiated to be formed from (a-3) 5 days after pulpotomy. Immunoreactivities of SVCT2 detected the newly differentiated osteoblast-like cells below the reparative dentin at (a-3) 5 and (a-4) 7 days after pulpotomy. (**b**) SVCT2 localization is recognized in the odontoblast-like cells 5 days after pulpotomy. (b-1 and b-4) SVCT2 staining (Green). (b-2) Nestin staining (odontoblast marker, red). (b-5) GLUT1 staining (Red) (b-3 and b-6) Merged images. The arrowheads indicate odontoblast-like cells that represented double positivity for SVCT2 and Nestin. (**c**) *Slc23a1* (cording SVCT2), *Slc2a1* (cording GLUT1), and *Nestin* mRNA expression in rat first molars after pulpotomy followed by MTA capping. Real time PCR used to quantify (c-1) *Slc23a1*, (c-2) *Slc2a1,* and (c-3) *Nestin* mRNA levels in normal first molar tissue and at each analyzed time point after pulpotomy. Data exhibit the mRNA expression levels of *Slc23a1*, *Slc2a1,* and Nestin normalized to *β-actin* mRNA level. Bars exhibit mean values ± standard error of the mean compared with controls (n = 3 for each group; only comparisons with *p*-value ≤ 0.05 are shown), Kruskal–Wallis test followed by Dunn's post hoc test. All mRNA levels peaked 5 days after pulpotomy. The closed star indicates the area exposed by a bur and capped with MTA. The asterisks indicate the odontoblast-like cell layer. The closed stars indicate the necrotic layer or reparative dentin layer. P, pulp. Scale = 20 μm.

**Figure 4:**
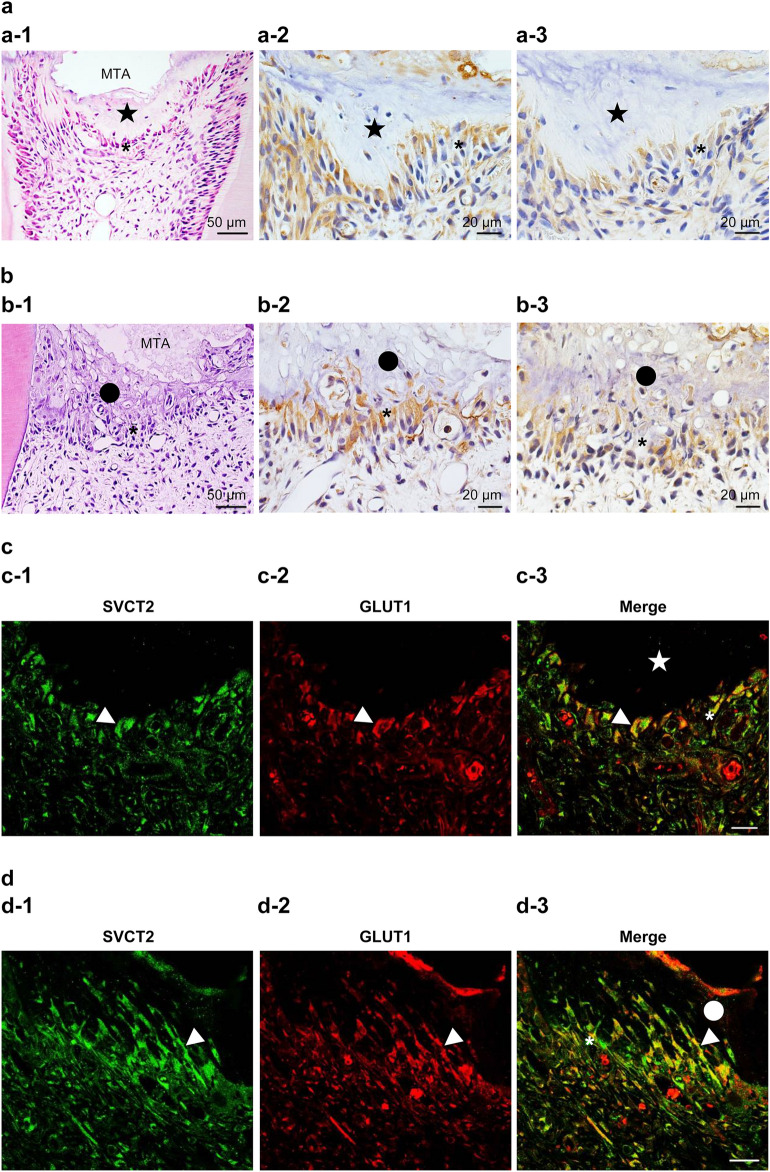
SVCT2 and GLUT1 are found with odontoblast-like cells in the pulp tissue of ODS rats after pulpotomy at 7 days followed by MTA capping. (**a** and **c**) ODS rats fed a normal diet (control; group 1). (**b** and **d**) ODS rats fed an ascorbic acid-free diet (group 2). (a-1 and b-1) Hematoxylin–eosin staining. Immunohistochemical staining of (a-2 and b-2) GLUT1 and (a-3 and b-3) SVCT2. (c and d) Double immunofluorescence staining of (c-1 and d-1, green) SVCT2 and (c-2 and d-2, red) GLUT1 in the cuboidal or columnar cells beneath the (**c**) reparative dentin or (**d**) necrotic layer. (c-3 and d-3) Merged images. The arrowheads indicate odontoblast-like cells that represented double positivity for SVCT2 and GLUT1. The closed star indicates the reparative dentin layer. The closed circle indicates the necrotic layer. The asterisks indicate the odontoblast-like cell layer. Scale = 20 μm.

**Figure 5:**
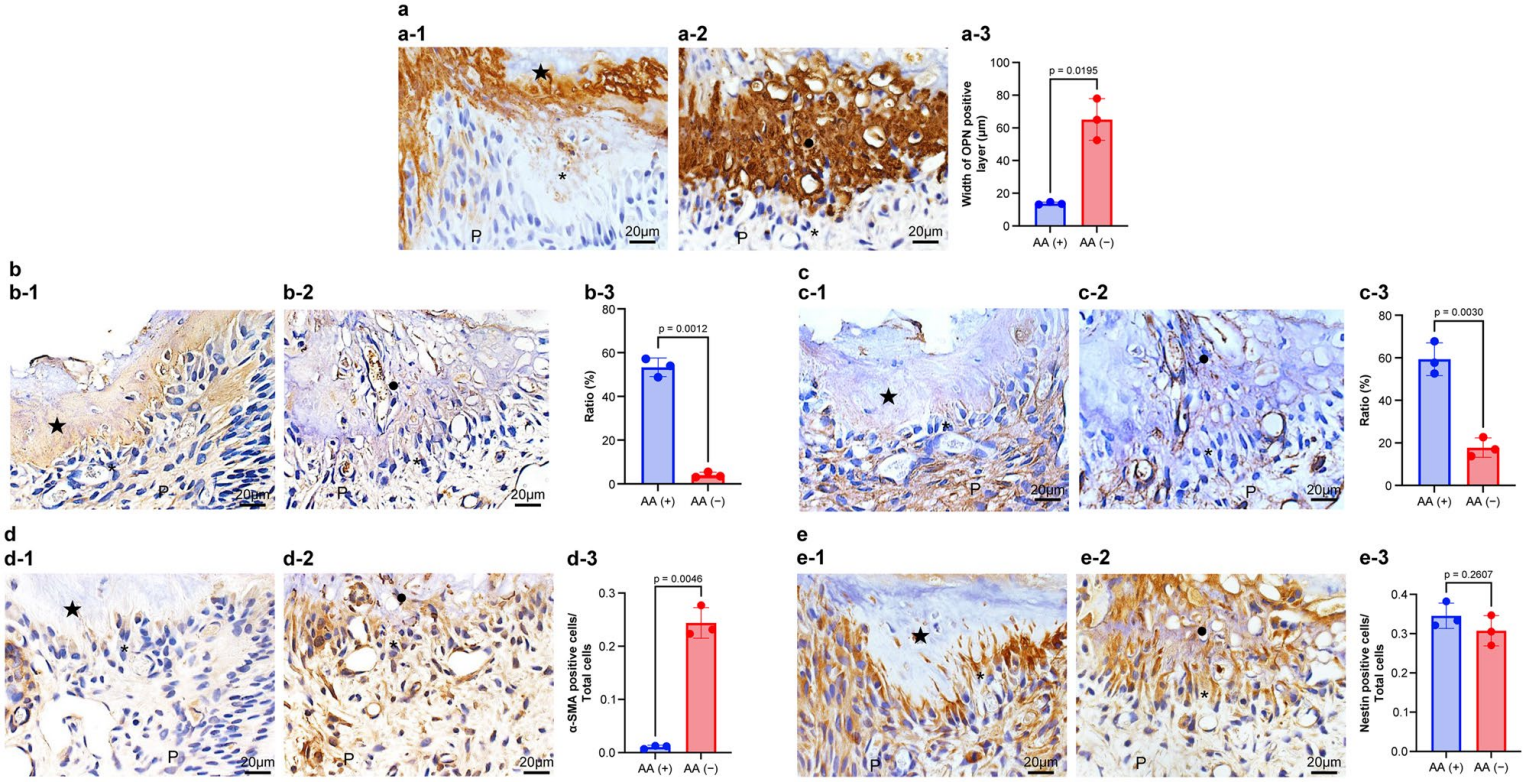
Immunohistochemical alteration of osteopontin (OPN), type I collagen (Col I), type III collagen (Col III), α-smooth muscle actin (α-SMA), and Nestin in the pulp tissue of ODS rat 7 days after pulpotomy followed by MTA capping. Immunohistochemical staining of (**a**) OPN, (**b**) Col I, (**c**) Col III, (**d**) α-SMA, and (**e**) Nestin. (a-1, b-1, c-1, d-1, and e-1) ODS rats fed a normal diet (control; group 1). (a-2, b-2, c-2, d-2, and e-2) ODS rats fed an ascorbic acid-free diet (AA deficiency: group 2). Data exhibit quantification of OPN, α-SMA, and Nestin (a-3, d-3, and e-3), or rations of Col I and Col III (b-3 and c-3). Bars exhibit mean values ± standard error of the mean compared with controls (group 1), (n = 3 for each group; only comparisons with *p*-value ≤ 0.05 are shown), Welch’s t-test. The OPN-immunopositive layer is observed above the reparative dentin in group 1 (a-1), whereas the thick OPN-positive (necrotic) layer is found in group 2 (a-2). The OPN layer of group 2 is thickened in the necrotic layer (a-3). The reparative dentin is positive for the Col I immunoreactivity of group 1 (b-1), although Col I immunoreactivity is diminished in the necrotic layer and the pulp tissue of group 2 (b-2). The Col I occupancy of group 2 is diminished in the reparative dentin (b-3). The pulp tissue beneath the pulpotomy site is positive for Col III of group 1 (c-1), although Col III immunoreactivity is diminished in the pulp tissue (c-2). The Col III occupancy of group 2 is diminished beneath the reparative dentin when compared with that in group 1 (c-3). Alpha-SMA immunoreaction is faint or almost none under the reparative dentin (d-1), although α-SMA-immunopositive cells of group 2 are observed beneath the necrotic layer (d-2). The α-SMA-immunopositive cells of group 2 are increased in odontoblast-like cells when compared with those in group 1 (d-3). Nestin-immunopositive cells are observed along the reparative dentin in group 1(e-1) or under the necrotic layer in group 2 (e-2). The Nestin-immunopositive cells are unchanged in odontoblast-like cells between groups 1 and 2 (d-3). The closed star indicates the reparative dentin layer. The closed circle indicates the necrotic layer.

**Figure 6:**
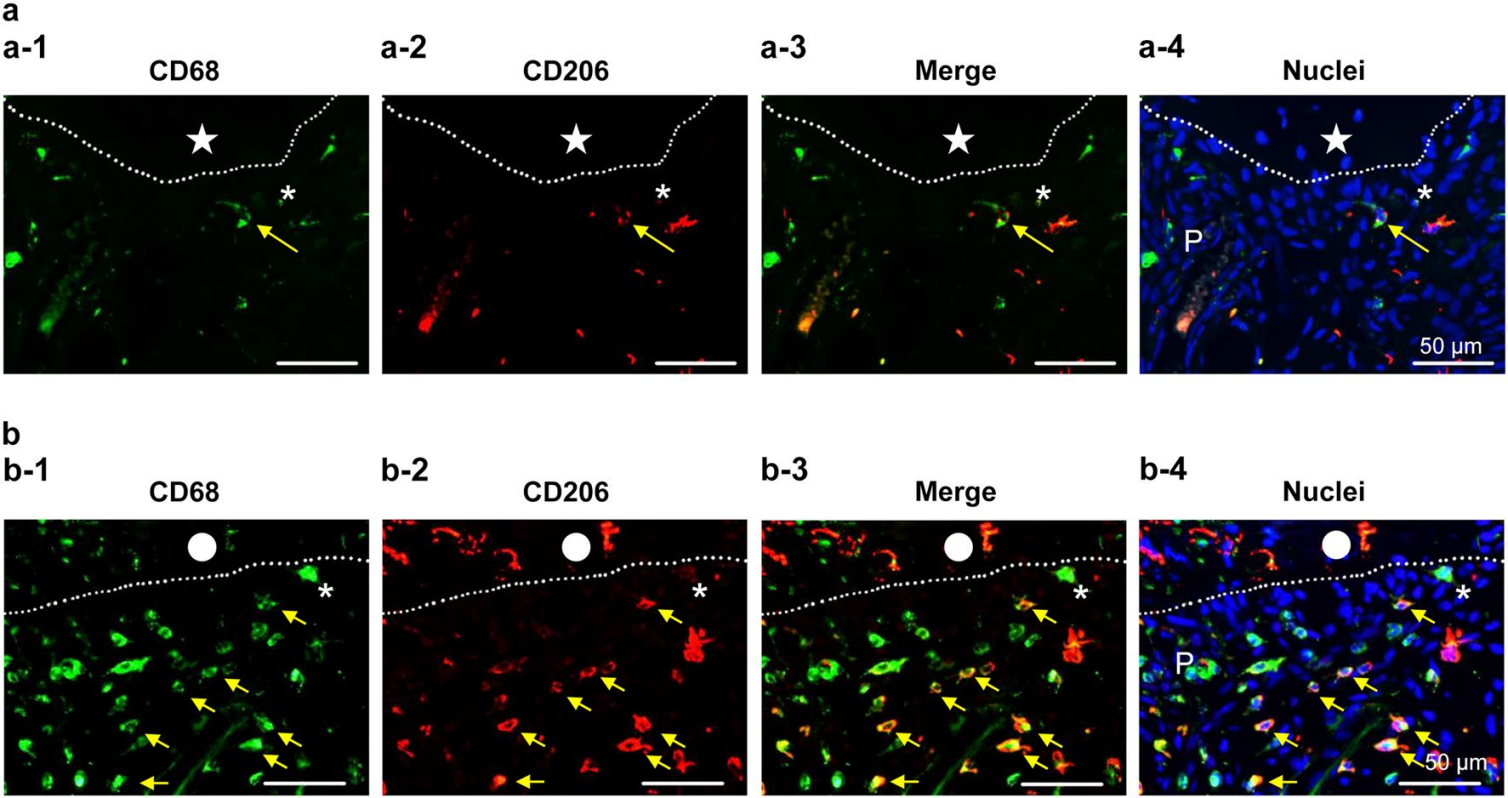
Immunofluorescence alteration of CD68 (green) and CD206 (red) in the pulp tissue of ODS rat 7 days after pulpotomy followed by MTA capping. Immunofluorescence of (**a**) ODS rats fed a normal diet (control; group 1), and (**b**) ODS rats fed an ascorbic acid-free diet (group 2). (a-1 and b-1) CD68 = green, (a-2 and b-2) CD206 = red, (a-3 and b-3) merged (a-1 or b-1) and (a-2 or b-2), (a-4 and b-4) nuclei = blue. CD68 positive cells in group 1 were diminished when compared with group 2. Moreover, the double-positive cells (CD68 and CD206), which indicated M2 macrophages, were also diminished in group 1. The dotted line indicates the border between the reparative dentin (**a**) or necrotic layer (**b**) and the pulp tissue. The closed star indicates the reparative dentin layer. The closed circle indicates the necrotic layer. The asterisk indicates the odontoblast-like cell layer. The yellow arrows indicate the M2 macrophages.

One day after surgery, SVCT2 immunoreactivity was not detected beneath the wound site, although odontoblasts other than those parts of the pulp tissue were positive for SVCT2 (Fig. 3a-1). At 3 and 5 days, some new odontoblast-like cells indicated SVCT2 immunoreactivity beneath the exposure site (Fig. 3a-2,a-3). Odontoblast-like cells are defined as the cells specifically observed beneath the wound healing site^12,13^. Especially, the ratio of SVCT2-immunopositive cells displayed a significant increase on day 5 (*p* = 0.0125) compared with that on day 1, but not on other days (*p* > 0.05) (Fig. 3a-5). Double immunofluorescence staining clearly overlapped SVCT2-positive on the odontoblast-like cells that showed Nestin-positive in the injured pulp tissue after 5 days (Fig. 3b-1–b-3). Additionally, we confirmed whether SVCT2-positive cells were consistent with the GLUT1-positive cells beneath the exposure site at 5 days. Double immunofluorescence staining clearly exhibited that SVCT2-postive overlapped with that of GLUT1 in odontoblast-like cells (Fig. 3b-4–b-6). At 7 days, perfect reparative dentin had formed, and newly differentiated odontoblast-like cells indicating SVCT2-positive in their cells were arranged beneath the reparative dentin (Fig. 3a-4).

Then, we analyzed how SVCT2 and GLUT1 mRNA expression changed over time by RT-PCR. We used Nestin as an odontoblast differentiation marker^11,14^. As shown in Fig. 3c, all mRNA levels examined on the operated side were significantly upregulated 5 days after surgery (*Slc23a2, p* = 0.0029; *Slc2a1*, *p* = 0.0086; Nestin, *p* = 0.0029) when compared with that on the contralateral side.

### Localization of the AA pathway in ODS rat molar pulp

To confirm how the injured part after pulpotomy follows in case of AA deficiency, we performed hematoxylin–eosin staining to detect the difference between group 1 (control: addition of AA) and group 2 (experimental: loss of AA). In group 1, we observed reparative dentin formation in the dental pulp. Additionally, we detected cells arranged beneath the reparative dentin (Fig. 4a-1). By contrast, in group 2, we observed necrotic layers beneath the wound surface instead of the reparative dentin. Moreover, we were unable to detect the arranged cells there (Fig. 4b-1).

Under conditions of AA deficiency, we analyzed whether the AA pathways are expressed in dental pulp similar to normal conditions during wound healing after pulpotomy. As shown in Fig. 4a-2,a-3,b-2,b-3, SVCT2 and GLUT1 are expressed in the cells beneath the reparative dentin or necrotic layer. Moreover, we confirmed whether SVCT2 and GLUT1 were expressed in the same odontoblast-like cells via immunofluorescence staining with anti-SVCT2 and anti-GLUT1 antibodies. SVCT2-positive cells were consistent with the GLUT1-positive cells in odontoblast-like cells (Fig. 4c,d, 4c: Gropu1, 4d: group 2).

### Effects of AA deficiency on reparative dentin formation

In this study, AA transporting pathways are present in both normal and AA deficiency during wound healing of pulp tissue. Then, we clarified what transpired beneath the wound surface using various antibodies on AA deficiency. A previous report demonstrated that immunocompetent cells and/or odontoblast-like cells deposit osteopontin (OPN) at the pulp–reparative dentin border to cause reparative dentin formation^15,16^. Additionally, OPN is crucial for the generation of type I collagen (Col I) by odontoblast-like cells during reparative dentin formation^17^. To confirm the relationship between OPN and Col I beneath the wound surface, we performed immunohistochemical staining with anti-OPN and anti-Col I antibodies in both situations of AA ingestion and deficiency. OPN immunoreactivity was detected beneath the wound surface in group 1 (controls) and group 2 (AA deficiency). However, the immunoreactive layer in group 2 (average thickness, 65.1 μm) was significantly thicker than that in group 1 (average thickness, 13.7 μm) (Fig. 5a, p = 0.0195). Regarding the ratio of Col I immunoreactivity occupancy, positive reactions were detected in the reparative dentin layer of group 1 (average occupancy, 53.3%) but hardly detected in group 2 (average occupancy, 4.02%) (Fig. 5b, p = 0.0012).

Type III collagen (Col III) is the first to be synthesized in the early phase of wound healing^18^. Essentially, Col III is also expressed in the pulp tissue as well as Col I^19^. We also performed immunohistochemical staining with anti-Col III antibody to confirm how expression changes are induced by AA deficiency. Col III immunoreactivity was detected in the pulp tissue beneath the reparative dentin layer in groups 1 and 2. However, the immunoreactivity in group 1 was increased when compared with that in group 2 (average occupancy: group 1, 59.4%; group 2, 17.8%) (Fig. 5c, p = 0.0030).

Alpha smooth muscle actin (α-SMA) is one of the markers for perivascular cells^20^. Additionally, α-SMA is expressed in myofibroblasts, which participate in dental pulp healing^21,22^. These findings indicate that the perivascular niche harbors α-SMA positive cells, which contribute to wound healing in the dental pulp. In our previous study, α-SMA and Nestin double-positive cells transiently assemble in the exposure site, and in the later stage, only the immunoreactivity of Nestin was expressed beneath the reparative dentin^21^. Thus, mature differentiated odontoblast-like cells express only Nestin, not α-SMA. In this study, α-SMA-positive cells were detected beneath the necrotic layer in group 2 (average ratio, 0.2438; Fig. 5d-2), whereas positive cells were barely detected beneath the reparative dentin of group1 (average ratio, 0.0106) (Fig. 5d, p = 0.0046). By contrast, we detected Nestin-positive cells beneath the reparative dentin in groups 1 and 2, with no significant difference (Fig. 5e).

OPN is mainly generated by macrophages in the early stage of wound healing of pulp tissue^15,23^. Especially, our previous study reported that M2 macrophage is induced in wound healing beneath the pulpotomy site^24^. The double-immunopositive cells of CD68 and CD206 can identify M2 macrophages^25^. We hypothesized that macrophages (M1 and M2) reside at the injured site and synthesize OPN there, as it is impossible to supply the collagen and repair the pulp tissue in group 2. Thus, it is very important to clarify the localization patterns of macrophages in both groups 1 and 2 after pulpotomy. Here we performed the double immunofluorescence of CD68 and CD208. Immunopositive cells of CD68 and double-immunopositive cells of CD68/CD206 in group 1 were clearly diminished when compared with those in group 2 (Fig. 6).

## Discussion

Here, we identified that rat dental pulp contains SVCT2 and GLUT1 for transporting AA, and that AA deficiency cannot induce the reparative dentin formation because of impaired collagen synthesis, impaired differentiation into odontoblast—like cell, and abnormal migration of M2 macrophages in wound area.

Reparative dentin is created by newly differentiated odontoblast-like cells at the exposure site, and metabolic activities of the cells increase^26^. We showed that the pulpal wound healing model using MTA is basically similar to that of our previous report^14^, that is, the primary healing process after MTA capping commonly demonstrated mild inflammatory and necrotic changes at the injured site (Fig. 3a1–a-3), followed by reparative dentin formation, which was first found in all specimens 7 days after treatment (Fig. 3a-4). Because damaged pulp tissue had nearly complete pulpal healing and reparative formation by 7 days, we focused on observing the reparative dentinogenesis under the MTA-capped area for 7 days to assess the effects of primary inflammation and repair in the case of AA deficiency.

**Figure 7:**
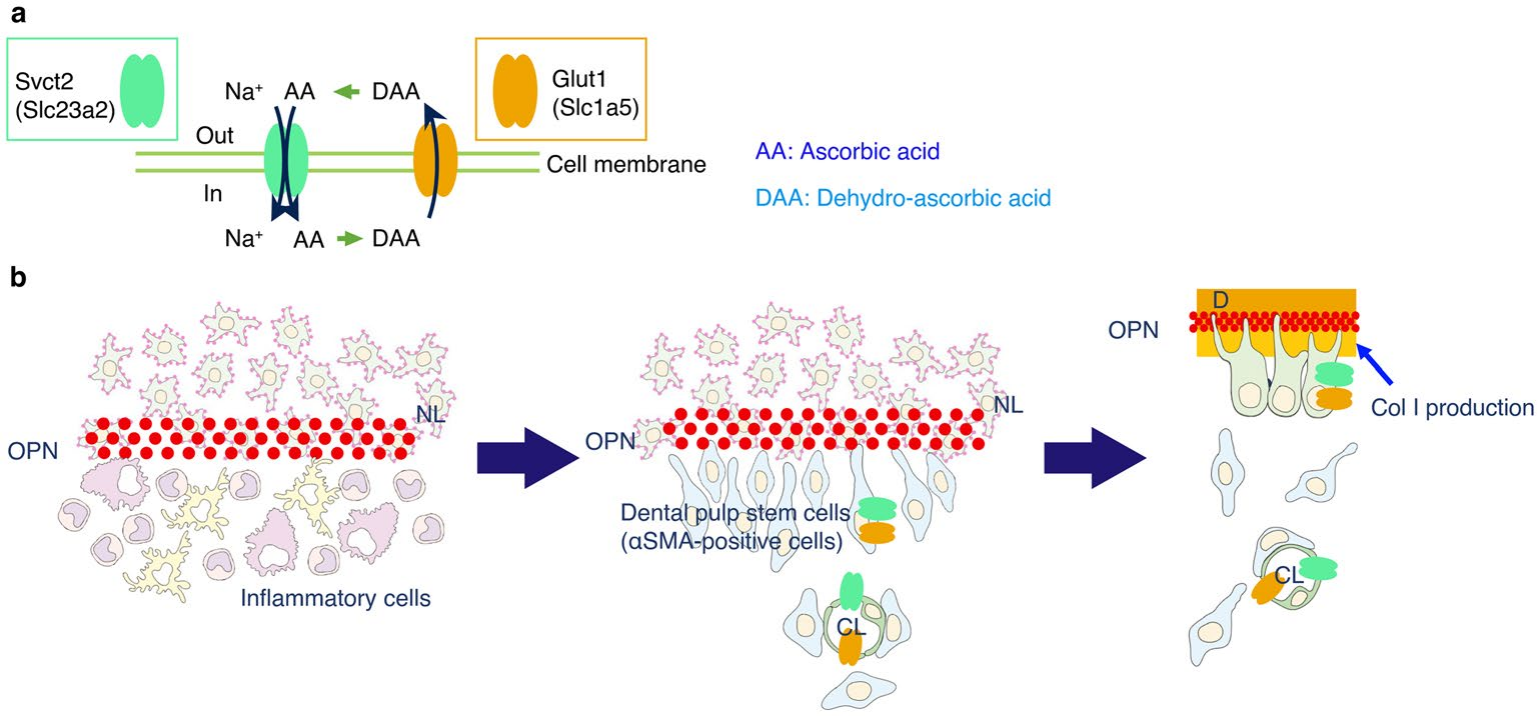
Schematic diagram of the putative relationship in the pulp–dentin border in the wound healing process after pulpotomy followed by MTA capping. AA, ascorbic acid; DAA, dehydro-ascorbic acid; OPN, osteopontin; NL, necrotic layer; OB, osteoblast; CL, capillary lumen; D, dentin (**a**). The pathway of the AA, (**b**) the role of the transporter of AA during wound healing. Immediately after pulp capping, OPN is deposited at the NL—inflammatory cell layer interface. Then, α-SMA-positive stem cells are recruited along the OPN layer. Finally, dental pulp stem cells differentiated the odontoblast-like cells, and subsequently, the odontoblast-like cells produced Col I to generate the reparative dentin. During the wound healing process in the dental pulp tissue, SVCT2 and GLUT1 might be involved in the differentiation of odontoblasts or wound healing in pulp tissue due to the supply of the AA. This figure was drawn by Naoto Ohkura using Illustrator 2022 (Adobe, San Diego, CA, USA).

Additionally, mature odontoblasts develop a characteristic autophagic system by reactive oxygen species^27^. SVCT2 is widespread and comparatively expressed in the body, where it contributes to the transport of AA to tissues with much demand for the vitamin for enzyme reactions or to prevent high metabolism activity cells or specific tissues from oxidative stress^28^. Thus, SVCT2 probably reflects the same roles in pulp tissue. This study provides new perceptions into the presumptive transporting pathway of AA in the whole pulp tissue during wound healing after pulpotomy. This study indicates that SVCT2 and GLUT1 are expressed in odontoblasts (including odontoblast-like cells), microvessels, and nerve fibers (Fig. 1b–d). In the choroidal plexus, AA is actively ingested in the cytoplasm via the SVCT2, and DHA is transported using GLUT1^29^. Taken together, these findings indicate that the SVCT2–GLUT1 pathway allows the widespread trafficking and/or circulation of AA in dental pulp tissue (Fig. 7a).

This study shows that *Slc23a2* (cording SVCT2), *Slc2a1* (cording GLUT1), and Nestin mRNA levels gradually increase in a similar time-dependent fashion during wound healing after MTA capping (Fig. 3c). Moreover, SVCT2 was localized in odontoblasts and odontoblast-like cells under the reparative dentin layer, similar to Nestin (Fig. 3b). Our previous study revealed that Nestin and GLUT1 were expressed in newly differentiated odontoblast-like cells in reparative dentin formation^11^. Furthermore, SVCT2 overexpression induces osteoblast differentiation, mineralization, and calcium deposition^30^. Therefore, our and other findings suggest that the transport of AA via SVCT2 may play a critical role in dentinogenesis and differentiation of odontoblasts because of the increase in metabolic activity and oxidative stress during wound healing in the pulp tissue.

It is difficult to carry substrates through the blood and/or various cells because of a cell wall barrier, and cell walls have a lipid bilayer. Endothelial cells, which obtain tight junctions, form the blood–nerve barrier (BNB)^31^ and specific transporters allow them to pass the substrates required by cells within the BNB. GLUT1 is localized in the cell membrane of endothelial cells and peripheral nerve fibers, including Schwann cells^11^. In this study, SVCT2 was also expressed in endothelial cells and nerve fibers in rat pulp tissue (Fig. 1c,d). These findings indicate that SVCT2 may be expressed at critical plasma membranes of the BNB and may arrange the crucial equipment for the carrier of AA across the cell membrane of vessels and peripheral nerve fibers. Moreover, SVCT2 plays a neuroprotective role during neural injury with ethanol^32^, suggesting that SVCT2 may be involved in recovery after microvessel and neuron injury.

OPN is a highly-phosphorylated glycoprotein that is one of the significant components of the bone extracellular matrix^33^ and is expressed in the dentin and the boundary between the necrotic layer and the tertiary dentin^34,35^. Col I is the main extracellular matrix protein in dentin, and it acts as a scaffold that accommodates minerals in the holes and pores of fibrils^36^. Presently, with the relationship between OPN and Col I during newly reparative dentinogenesis, macrophages and dendritic cells generate OPN to lead to odontoblast-like cell differentiation, and next immature odontoblast-like cells secrete OPN to increase the production of Col I^17^. Regarding the generation of the reparative dentin in ODS rats (AA-deficient rats), the OPN immunoreactive layer was thickly deposited (Fig. 5a-1 and a-2), and Col I immunoreactivity was not detected under that layer (Fig. 5b-1 and b-2). OPN at the necrotic layer suggests that it plays a crucial role in the stem cells/progenitors’ migration to the wound site and their differentiation into odontoblast-like cells during reparative dentin formation^2^. Additionally, these findings suggest that OPN deposition is one of the triggers of Col I production in immature odontoblast-like cells and that Col I may be essential for the terminal differentiation into dentin-forming cells (Fig. 7b). However, in ODS rats (AA deficiency), immature odontoblast-like cells may continue the production of OPN but not of Col I; thus, they cannot differentiate into mature cells. Moreover, previous reports have demonstrated that macrophages and dendritic cells secrete OPN in the injured pulp^16^.

Macrophages act as guards that protect the immune system^37^. Especially, M2 macrophages control inflammatory responses (e.g., debris scavenging, acceleration angiogenesis, tissue remodeling, and repair)^38–40^. M2 macrophages are also involved in pulp repair after MTA pulpotomy^24^. We demonstrated that both total macrophages and M2 macrophages in group 2 (AA deficiency) increased when compared with group 1 (controls) (Fig. 6), suggesting that AA deficiency may cause persistent inflammation and delayed healing in the pulp tissue; subsequently, many macrophages accumulated beneath the wound site, which might continue to synthesize OPN. Additionally, M2 macrophages may not function sufficiently as modulators of inflammation and/or repair in the pulp tissue without AA.

Col III is the second most abundant collagen type in the human body^41^ and an important component of fibrillar collagen organization. Col III frequently assembles with Col I to form heterotypic type I/III fibrils^42^ and is involved in collagen cross-linking^43^. As mentioned above, Col III is the first to be synthesized in the early phase of wound healing^18^. Other researchers showed that Col III is present in the pulp similar to Col I^18^. We found that clearly poorer expression of Col III in group 2 (AA deficiency) when compared with that of group1 (controls) (Fig. 5c), suggesting that AA deficiency impaired the synthesis of Col III that is required to repair the injured pulp area.

Nestin is expressed in mature and immature odontoblast-like cells^15,44^. α-SMA, which is a myofibroblast marker, is also localized in immature odontoblast-like cells but not in mature odontoblast-like cells during wound healing in pulp tissue^21,22^. In the case of AA supply, the relationship between Nestin- and α-SMA-positive cells are revealed in cells beneath the wound healing but not AA deficiency (Fig. 5). These findings suggest that AA is crucial to the terminal differentiation of odontoblast-like cells. A previous study supports the notion that AA is essential to differentiate mesenchymal stem cells and osteoblasts^45^.

Previous studies have reported that antioxidants suppressed inflammation and showed simultaneously concentrated collagen deposition during wound healing in rat skin^46–49^. Because AA is also one of the antioxidants and AA induced condensed collagen deposition (Fig. 5b,c), it may possess a similar effect.

AA likely contributes to all phases of wound healing regarding apoptotic cells, antioxidant protection, collagen synthesis, and bone formation^50^. AA is required for the hydroxylation of proline residues in procollagen, and hydroxyproline stabilizes the collagen triple helical structure^51^. That is, AA plays a crucial role in the generation of collagen during wound healing. A previous report demonstrated that AA deficiency may affect macrophage random migration functions and could impair parameters of host defenses effective against microbial infections^52^. Moreover, AA is required for timely neutrophil apoptosis and clearance in the inflammatory phase^53^. The use of initial high-dose AA supplements appears to be useful in wound healing as plasma and tissue levels are rapidly depleted in response to wounding^54^. However, the oral administration of AA is not harmful to patients due to the strict control of its absorption and urinary excretion^54^. Concretely, as oral administration exceeds 200 mg, the relative absorption diminishes, excretion in urine increases, and subsequently, the bioavailability of AA diminishes^55^. Thus, the oral administration plan should be constructed carefully if we use AA during wound healing.

A limitation of this study is that the wound healing grade was evaluated with only the minimal AA concentration required for survival. As mentioned above, high-dose AA supplements appear to be useful in wound healing^48^. Considering the use of high-dose AA for wound healing, we speculate that it is necessary to determine the optimal concentration for maximal efficacy after pulpotomy. This possibility can be the aim of future studies.

In conclusion, SVCT2 immunoreactivities were detected in odontoblasts, endothelial cells, and peripheral nerve fibers. The spatiotemporal changes in mRNA expression and localization of SVCT2/GLUT1 were observed in pulp tissue after pulpotomy. Additionally, in ODS rats (AA deficiency), α-SMA-positive cells (myofibroblasts) may not have differentiated into odontoblast-like cells that eventually produce Col I to form reparative dentin. These findings provide novel insights into the pathways and roles of AA via SVCT2 and GLUT1 in the pulp tissue and may be useful in advancing new therapeutic strategies for dental therapy.

## Methods

All animal experiments were conducted in compliance with the protocol reviewed by the Institutional Animal Care and Use Committee of Niigata University and approved by the President of Niigata University (Permit No. 28-312-1). The study was conducted in accordance with the relevant ARRIVE (Animal Research: Reporting of in Vivo Experiments) guidelines and regulations.

In this study, we used 8-week-old male specific pathogen-free Wistar rats (n = 27, Charles River, Yokohama, Japan) and six 8-week-old male ODS rats (n = 6, ODS/Shi Jcl-od/od; CLEA Japan Inc., Tokyo, Japan). We provided Supplementary Table 1, showing the number of animals used in each experiment. The sample size was determined based on previous literature and our previous experience to give sufficient standard deviations of the mean so as not to miss a biologically important difference between groups.

They were raised in plastic cages in an animal room with a 12 h light/dark cycle, and an ambient temperature was maintained at 25 °C. All Wistar rats took standard pellet chow and water ad libitum. As described above, ODS rats cannot produce AA in the body because they lack the gene for AA synthesis.

ODS rats were divided into two groups. In group 1 (controls), they were not subjected to dietary change, and they received water containing 1 mg/mL of AA. The total supplement of AA/kg is adequate for maximum growth and protection of scurvy in group 1 (controls)^56^. In group 2 (AA deficiency), the rats received a diet without l-ascorbic acid (CL-2; Nihon CLEA Japan Inc.) instead of the normal diet^56^.

### Pulpotomy procedures

Pulpotomy procedures were described previously^14^. Concretely, the rats were anesthetized intraperitoneally with a solution containing medetomidine hydrochloride 0.375 mg/kg, midazolam 2 mg/kg, and butorphanol tartrate 2.5 mg/kg. Under anesthesia with an intraperitoneal injection, the occlusal surface of the upper left first molar was exposed with a #1 round carbide bur (ISO No. 1/008; diameter, 0.8 mm). The exposed pulp was washed with 5% sodium hypochlorite (Neocleaner; Neo Dental Chemical Products, Tokyo, Japan), followed by rinsing with sterile saline. Bleeding was arrested by sterile cotton pellets. The exposed area was capped with MTA (white ProRoot MTA; Dentsply Tulsa Dental, Tulsa, OK), mixed with sterile saline according to the manufacturer's protocol. MTA was put on the injured site, and the cavity was filled with a flowable composite resin (Beautifil Flow; Shofu, Kyoto, Japan). These prepared samples were used for immunohistochemical staining and gene expression analyses. The contralateral maxillary first molar of the same animal was used as the control and/or normal. The observation periods were 1, 3, 5, 7, and 14 days after treatment (1, 3, 5, and 7 days: n = 3 each for immunohistochemistry, 1, 3, 5, 7, and 14 days: n = 3 each for gene expression analyses, 7 days: n = 3 each group for ODS rat analyses).

### Immunohistochemical staining

Immunohistochemical staining was described previously^14^. Concretely, after pulpotomy, the animals received transcardiac perfusion of 0.1 M phosphate buffer (pH 7.4) and then fixed by 4% paraformaldehyde (PFA) for 10 min. The pulpectomized tooth were extracted en bloc with surrounding tissue and submerged in 4% PFA for another 24 h. After demineralization in a 10% ethylenediaminetetraacetic acid solution for 4 weeks at 4 °C, the specimens were embedded in paraffin, and they were sectioned sagittally at 4-μm thickness and subjected to immunohistochemistry.

Sections were deparaffinized with lemosol (Wako, Osaka, Japan) and then hydrated via sequential immersion in 100%, 95%, 90%, 80%, and 70% alcohol solutions. Subsequently, antigen retrieval was performed with 10 mmol/L citric acid buffer (pH 6.0). For 3,3′-diaminobenzidine (DAB) staining, sections were treated with methanol containing 3% hydrogen peroxide (Wako) in order to block endogenous peroxidase activity.

Supplementary Table 2 shows the primary and secondary antibodies used. The secondary antibodies were horseradish peroxidase (HRP)-labeled swine antirabbit IgG (1:200), HRP-labeled rabbit antimouse IgG (1:200), HRP-labeled rabbit antigoat IgG (1:200, Dako, Glostrup, Denmark), Alexa Fluor 488 goat antirabbit IgG, Alexa Fluor 488 donkey antigoat IgG, Alexa Fluor 546 goat antimouse IgG, and Alexa Fluor 546 donkey antimouse and antirabbit (1:200, Thermo Fisher Scientific).

The immunohistochemical stainings of SVCT2, GLUT1, Nestin, OPN, α-SMA, Col I, and Col III were performed overnight reaction to a primary antibody at 4 °C, and then reacted the corresponding to secondary HRP-labeled antibody for 1 h at 25 °C. Finally, sections were stained by a DAB substrate kit (Dako), counterstained with hematoxylin, and observed microscopically. Digital images were taken with a CCD camera attached to a microscope (Eclipse E800; Nikon, Tokyo, Japan).

For double or triple immunofluorescence staining, the activated sections were washed with PBS and then reacted overnight at 4 °C with primary antibodies against SVCT2, GLUT1, RECA1, PGP9.5, Nestin, CD68, and CD206 antibodies used to verify endothelial cells, peripheral nerve fibers, odontoblasts, pan-macrophages, and M2 macrophages, respectively. Nestin, a useful marker of odontoblast, is also detected in the newly differentiated odontoblast-like cells following direct pulp capping with MTA^11,15^. After rinsing with PBS, the sections were further reacted for 1 h at 4 °C using a cocktail of secondary antibodies. 4′6-Diamidino-2-phenylindoledihydrochloride (DAPI; ProLong Diamond Antifade Mountant with DAPI; Thermo Fisher Scientific) was used for nuclei counterstaining. Negative controls were prepared by adding the PBS instead of the primary antibodies. These controls did not detect any specific immunoreactivity (data not shown). Digital images were taken with a CCD camera attached to a confocal laser scanning microscope (IX71; Olympus, Tokyo, Japan) or an epifluorescence microscope (Eclipse E800; Nikon). Images of the same field stained with different fluorochromes were superimposed using image processing software (Photoshop CC 23.4; Adobe, San Diego, CA, USA) to create double/triple color images.

With reference to our previous study^24^, the regions to be analyzed were determined within 100 μm from the wound surface. The zone area, thickness, or cell number of each immunoreactivity by the specific antibodies was analyzed using Image J (version 1.50i, NIH, Bethesda, MD, USA)^57^. Regarding the OPN quantification, three points were randomly selected from the reparative dentin layer, and the average value of their thicknesses was used. Quantification of Col I and Col III was obtained from the following formula:

Percentage of Col I-immunopositive area = Col I-immunopositive area in the reparative dentin layer/total reparative dentin layer

Percentage of Col III-immunopositive area = Col III-immunopositive area in determined analysis area/total determined analysis area

Then, the ratio of immunopositive cells (α-SMA and Nestin) in each region was calculated by dividing the ‘‘immunopositive cell number’’ by the ‘‘total nuclei.’’.

### Gene expression analyses

Gene expression analyses were described previously^14^. Concretely, total RNA was extracted from the first molar with a TRIzol reagent (Thermo Fisher Scientific, MA, USA) after cutting all roots, according to the manufacturer’s procedure. Single-strand cDNA was produced from 0.5 μg of total RNA with reverse transcriptase using a PrimeScript RT Master Mix (Perfect Real Time; Takara Bio Inc., Otsu, Japan). PCR was performed by a gene amplification system (GeneAmp PCR system 9700; Applied Biosystem, Foster City, CA, USA) using specific primers for SVCT1 and SVCT2.

Quantitative RT-PCR was performed with a GeneAmp PCR system 7900 HT (Applied Biosystems, Foster City, CA, USA) with SYBR Premix Ex Taq II (Perfect Real Time; Takara Bio Inc.), according to the manufacturer’s protocol. A standard curve was produced for untreated first molars, and, subsequently, quantified the amount of specific mRNA in each sample. The initial mRNA content of cells was normalized by the amount of *β-actin*. PCR was performed using specific primers for rat *Slc2a1* (cording GLUT1), *Slc32a2* (cording SVCT2), *Nestin*, and *β-actin*. Supplementary Table 3 shows the sequences of all primers.

### Statistical analysis

The Kruskal–Wallis test followed by Dunn’s post hoc test was used to perform statistical comparisons among multiple groups. If there were only two groups, unpaired two-tailed *t*-test (Weltch’s *t*-test) was used. The threshold for statistical significance was set to be *p* < 0.05. All computations were conducted with GraphPad Prism 9 (GraphPad Software, Inc., San Diego, CA, USA).

## Supplementary Information

Supplementary Information.

## Data Availability

All data created or measured during this study are included in this article and its supplementary information files.
