# Supplementary material for: SVCT2-GLUT1-mediated ascorbic acid transport pathway in rat dental pulp and its effects during wound healing
Source: Sci Rep. 2023 Jan 23;13:1251. doi: 10.1038/s41598-023-28197-9 (PMC9870884; doi:10.1038/s41598-023-28197-9)
Supplement: Supplementary file 1 — Supplementary Information. [file 41598_2023_28197_MOESM1_ESM.docx]

**SVCT2-GLUT1-mediated ascorbic acid transport pathway in rat dental pulp and its effects during wound healing**

*Naoto Ohkura^1^, Kunihiko Yoshiba^2^, Nagako Yoshiba^1^, Naoki Edanami^1^, Hayato Ohshima^3^, Shoji Takenaka^1^, Yuichiro Noiri^1^

Affiliations

^1^Division of Cariology, Operative Dentistry and Endodontics, Department of Oral Health Science, Niigata University Graduate School of Medical and Dental Sciences, Niigata, Japan.

^2^Division of Oral Science for Health Promotion, Department of Oral Health and Welfare, Niigata University Graduate School of Medical and Dental Sciences, Niigata, Japan

^3^Division of Anatomy and Cell Biology of the Hard Tissue, Department of Tissue Regeneration and Reconstruction, Niigata University Graduate School of Medical and Dental Sciences, Niigata, Japan.

*Correspondence: [ohkura@dent.niigata-u.ac.jp](mailto:ohkura@dent.niigata-u.ac.jp)

**Supplementary Figure 1.**


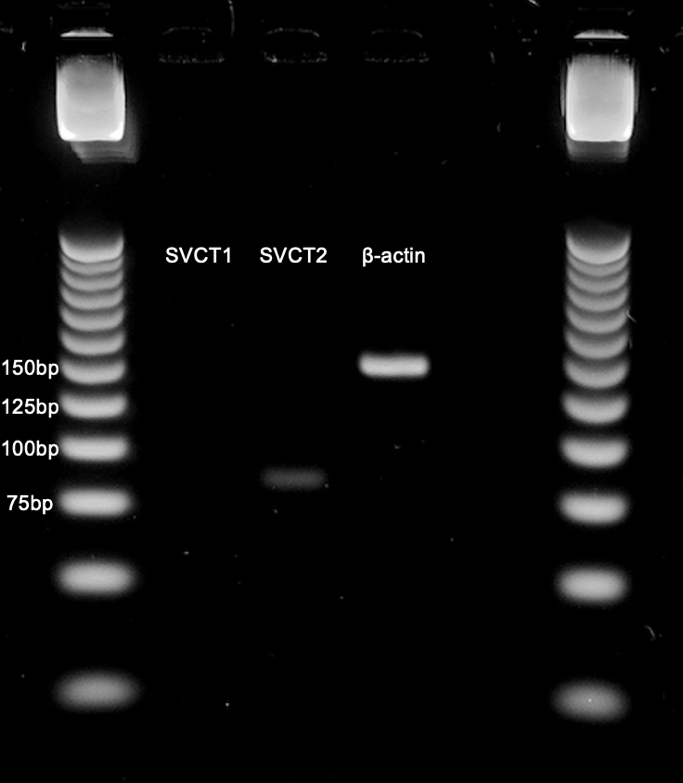


Expression of sodium-dependent vitamin C transporters (SVCTs) mRNA with RT-PCR in rat molar dental pulp. This picture shows a full-length gel from Figure 1a.

**Supplementary Table 1. Number of rats used in this study**

| Wistar Rats |  |  |  |  |
| --- | --- | --- | --- | --- |
| 1 day after pulpotomy | 3 days after pulpotomy | 5 days after pulpotomy | 7 days after pulpotomy | 14 days after pulpotomy |
| Immunostaining, 3 | Immunostaining, 3 | Immunostaining, 3 | Immunostaining, 3 | Immunostaining, 0 |
| Real-time PCR, 3 | Real-time PCR, 3 | Real-time PCR, 3 | Real-time PCR, 3 | Real-time PCR, 3 |
|  |  |  |  |  |
| ODS rats |  |  |  |  |
| 7 days after pulpotomy |  |  |  |  |
| Group 1 (AA＋), 3 |  |  |  |  |
| Group 2 (AA−), 3 |  |  |  |  |

**Supplementary Table 2. Antibodies used in this study**

| **Antibody** | **Clone** | **Dilution** | **Source** |
| --- | --- | --- | --- |
| Goat anti-SVCT2 | G-19 | 1: 50 | Santa Cruz Biotechnology, Dallas, TX, USA |
| Rabbit anti-GLUT1 | Plolyclonal | 1: 100 | Proteintech, Rosemont, IL, USA |
| Mouse anti-Nestin | Rat-401 | 1: 100 | Millipore, Darmstadt, Germany |
| Rabbit anti-OPN | O-17 | 1 µg/mL | Immuno-Biological Laboratories, Gumma, Japan |
| Mouse anti-α-SMA | 1A4 | 1: 100 | Sigma-Aldrich, St Louis, MO, USA |
| Rabbit anti-Col I | Polyclonal | 1: 100 | Abcam, Cambridge, United Kingdom |
| Rabbit anti-Col III | Polyclonal | 1: 100 | Abcam, Cambridge, United Kingdom |
| Mouse anti-CD68 | ED-1 | 1: 250 | Novus Biologicals, Centennial, CO, USA |
| Rabbit anti-CD206 | Polyclonal | 1: 1000 | Abcam, Cambridge, United Kingdom |
| Mouse anti-RECA-1 | HIS52 | 1: 50 | AbD Serotec, Oxford, UK |
| Rabbit anti-PGP9.5 | Polyclonal | 1: 300 | UltraClone Limited, UK |

**Supplementary Table 3. Primers used in this study**

| **Molecular** | **Accession Number** | **Primer** | | |
| --- | --- | --- | --- | --- |
| **name** |  | **Name of oligomers** | **Sequence** | **Expected size** |
| ***Slc2a1*** | NM_138827.1 | Sense primer | tggctttgtggccttctttg | 140bp |
|  |  | Anti-sense primer | tgcccacgatgaagtttgag |  |
| ***Slc32a1*** | NM_031782 | Sense primer | ttcggcttgctccattctac | 74bp |
|  |  | Anti-sense primer | aaacaggcaacaggaaggtg |  |
| ***Slc32a2*** | NM_017316 | Sense primer | cggaacctatttgtgcttgg | 84bp |
|  |  | Anti-sense primer | gcctgtaaccagagggttttg |  |
| ***Nestin*** | AF538924.1 | Sense primer | tccttagccacaaccctcaac | 109bp |
|  |  | Anti-sense primer | agatttgcccctcatcttcc |  |
| ***Beta-actin*** | NM_031144.2 | Sense primer | cagggtgtgatggtgggtat | 146bp |
|  |  | Anti-sense primer | gtgtggtgccaaatcttctc |  |
